# Overview of research on virus-resistant breeding of melon

**DOI:** 10.3389/fpls.2024.1500246

**Published:** 2024-12-12

**Authors:** Shoubo Tian, Qiannan Diao, Yanyan Cao, Dongwei Yao, Wenxian Zhang, Hui Zhang, Xuan Du, Yongping Zhang

**Affiliations:** ^1^ Horticultural Research Institute and Shanghai Key Lab of Protected Horticultural Technology, Shanghai Academy of Agricultural Sciences, Shanghai, China; ^2^ Shanghai Agriculture Technology Extension and Service Center, Shanghai, China

**Keywords:** virus- resistance, *Cucumis melo* L., QTL, MAS, sustainable agriculture

## Abstract

The development of virus-resistant melon varieties not only poses challenges in balancing melon quality and resistance but also contributes to sustainable agricultural development. This research focuses on the exploration and application of various breeding techniques to enhance the virus resistance of melon varieties. Molecular markers associated with virus resistance genes have been identified and utilized in marker-assisted selection, enabling more efficient and targeted breeding. Genetic engineering approaches have also shown promise, introducing specific resistance genes into melon genomes. In addition, traditional breeding methods, such as hybridization and selection, continue to play an important role in creating virus-resistant melon lines. The combination of these approaches holds great potential for developing melon varieties with improved virus resistance, thereby increasing yield and quality, and reducing the economic losses caused by viral infections in melon production.

## Introduction

Breeding virus-resistant melon (*Cucumis melo* L.) varieties is an essential area of agricultural research aimed at mitigating the significant economic losses caused by various viral infections. Traditionally, efforts to develop such resistant traits are faced numerous challenges, including the complex and polygenic nature of virus resistance and the intricate interactions between viruses, host plants, and the environment (Argyris et al., 2015). Traditional breeding methods often proved insufficient due to these complexities and the time-intensive processes involved (Kenney et al., 2020; Kesh and Kaushik, 2021). Traditional plant breeding of melon has been carried out for 100 years, among which plants have initially cultivated pollinated varieties. Recently, in the past 30 years, the improvement of melon has been completed by more traditional hybridization techniques. The improvement of germplasm is relatively slow and limited by gene pool (Nuñez-Palenius et al., 2008).

Recent advancements in molecular biology have revolutionized melon breeding programs. The application of quantitative trait loci (QTL) mapping and molecular marker-assisted section(MAS has dramatically improved the efficiency and precision of developing virus-resistant melon varieties. These modern genetic techniques allow for the identification and incorporation of specific hereditary traits linked to disease resistance, significantly reducing the breeding cycle time compared to traditional methods (Pérez-de-Castro et al., 2020; Shahwar et al., 2023). Discoveries such QTL associated with resistance to Melon leaf curl New Delhi virus that impacted melon crops across Europe, Asia, and other regions have been pivotal in combating viral diseases (Lecoq and Katis, 2014; Siskos et al., 2022).

Despite these scientific advancements, developing virus-resistant melons continues to present challenges. Viruses can rapidly evolve, potentially overcoming resistance traits introduced through breeding programs. Furthermore, achieving the balance between virus resistance and maintaining other desirable agronomic traits, such as fruit quality and yield, remains a complex task for breeders. The integration of resistance traits must be managed carefully to avoid negative impacts on the overall genetic background of commercial melon varieties (Argyris et al., 2015; Maachi et al., 2022).

The importance of breeding virus-resistant melons extends beyond economic considerations; it is also crucial for sustainable agriculture and environmental conservation. Reducing reliance on chemical insecticides for virus control, through the development of resistant melon varieties, contributes to ecological balance and minimizes environmental pollution. Ongoing research and future directions, including genome re-sequencing and genome-wide association studies (GWAS), continue to hold promise for further advancements in this field, ensuring the development of resilient melon cultivars capable of withstanding viral threats while meeting commercial standards (Desbiez and Lecoq, 2004; Castle et al., 2017).

## Viruses and melons

Melons are susceptible to a variety of viral diseases that can significantly impact crop yield and quality (Shahwar et al., 2024) and each with distinct characteristics and effects on the plants. The spread of crop viral pests has increased dramatically in recent years due to globalization, trade, and climate change among other factors (Nicaise, 2014; Jeong et al., 2024). Virus diseases in crops are responsible for enormous yield losses globally, estimated to cost more than $30 billion annually (Nicaise, 2014; Rabadán et al., 2023). The economic impact underscores the importance of developing and implementing effective virus resistance breeding programs in melons and other crops.

Melons are susceptible to a wide range of viruses, with more than 35 different viruses reported to affect cucurbits, including significant pathogens like the cucurbit aphid-borne yellows virus (CABYV), ToLCNDV, and Watermelon Mosaic Virus (WMV) (Maachi et al., 2022). These viral infections severely impact the quality and yield of melons, leading to substantial economic losses (Maachi et al., 2022; Siskos et al., 2022). Several viruses are known to infect melon crops, including Cucumber Mosaic Virus (CMV), Squash Mosaic Virus (SqMV), Zucchini Yellow Mosaic Virus (ZYMV), and Watermelon Mosaic Virus (WMV) (Lecoq and Katis, 2014). Among these, WMV and Cucurbit aphid-borne yellows virus (CABYV) are particularly prevalent in melon crops in certain regions (Maachi et al., 2022). WMV, also referred to as Marrow mosaic virus or Melon mosaic virus, is part of the Potyviridae family and affects many different other plants (Argyris et al., 2015). Genetic analyses have shown that the WMV population in Spain is highly homogeneous, consisting of a single pathotype closely related genetically (Desbiez and Lecoq, 2004). Viruses such as CABYV and WMV can threaten melon production by serving as reservoirs in weed species surrounding melon fields, ensuring virus survival from one season to the next (Gur et al., 2017). These viruses have been detected consistently in commercial melon production areas, indicating the significant role of weeds in the virus life cycle (Gur et al., 2017).

New viruses continue to emerge and pose threats to melon production. For example, Melon necrotic spot virus (MNSV) has been observed in multiple regions and is known to cause significant yield losses (Gomez-Aix et al., 2016). Similarly, Melon leaf curl New Delhi virus is creating substantial yield losses throughout Europe and Asia (Kesh and Kaushik, 2021). Identifying and breeding for resistance to these emerging viruses is essential for maintaining sustainable melon production.

By understanding the diverse range of viruses that affect melons, their impact on the crop, and the mechanisms of natural resistance, researchers and breeders can develop more resilient melon varieties that can withstand viral diseases and ensure stable crop yields. Infection with viruses such as CMV affects melon physiology by altering sugar transport and carbohydrate levels. CMV-infected melon leaves show high concentrations of reducing sugars and low starch levels, indicating a disruption in the plant’s normal metabolic processes (Shalitin and Wolf, 2000).

Several melon accessions have been identified as valuable sources of virus resistance genes. For instance, some melon varieties are resistant to Cucurbit Yellow Stunting Disorder Virus (CYSDV), which has been spreading through North America, Europe, and North Africa (Pérez-de-Castro et al., 2020). And *C. melo* var. *cantalupensis* accessions such as PMR 45, PMR 5, PMR 6, and WMR 29, as well as *C. melo* var. *momordica* accessions PI 124111, PI 124112, and PI 414723, have been widely used in breeding programs due to their multiple resistance genes (Cui et al., 2022). Understanding and utilizing these natural resistances are crucial for developing virus-resistant melon cultivars.

## Breeding methods for virus resistance in melons

Breeding for virus resistance in melons is a critical area of agricultural research due to the substantial economic impact of viral diseases on melon crops. Traditional breeding methods, marker-assisted selection (MAS), and genetic engineering have all been employed to develop virus-resistant melon lines.

Traditional breeding has played a significant role in the genetic improvement of melons for virus resistance. These methods typically involve selecting plants with desirable traits, such as resistance to viruses, and cross-breeding them to produce offspring that inherit these traits. However, traditional breeding can be limited by factors such as sexual incompatibility between different species or genera, and the time-consuming nature of the breeding process (Kesh and Kaushik, 2021). In the case of melons, plants are often infected by complexes of phylogenetically distinct viruses, making it challenging for resistance traits generated through traditional breeding to be economically viable (Kenney et al., 2020). One traditional breeding method that has been modified for improved efficiency is single-seed descent. This method rapidly develops inbred lines through self-pollination and is particularly effective for improving quantitative traits such as yield and earliness (Samantara et al., 2022).

Marker-assisted selection (MAS) is a molecular breeding technique that uses DNA markers linked to desirable traits to select plants with those traits during the breeding process. MAS enhances the precision and efficiency of traditional breeding methods by allowing breeders to identify plants with virus resistance genes more accurately and at earlier stages of development (Collard and Mackill, 2007). The development of melon lines such as ME8094, which show resistance to multiple viruses, has been facilitated by the use of molecular markers linked to QTL associated with virus resistance (Bachiava et al., 2018). MAS has proven to be particularly useful in melon breeding, as it can help overcome the challenges associated with traditional breeding methods. For instance, resistance loci can be identified and introgressed into elite cultivars without the need for extensive phenotypic selection (Collard and Mackill, 2007).

In addition to MAS, genetic engineering is a widely used technology for developing virus-resistant melon plants. The relatively small melon genome, comprising approximately 375 Mbp and 27,427 protein-coding genes, has been sequenced, providing valuable insights into genetic modifications that can enhance virus resistance (Shahwar et al., 2023). Genetic engineering allows for the direct manipulation of the melon genome to introduce virus resistance genes, offering a more targeted and efficient approach compared to traditional breeding methods.

Genetic mapping of the virus resistant sources has led to the identification of numerous dominant and recessive genes, as well as QTL, conferring resistance to viruses like powdery mildew and downy mildew (Cui et al., 2022). The continuous improvement in breeding methods, coupled with advances in molecular biology, holds promise for the future development of melon cultivars with enhanced virus resistance.

## Challenges in developing virus-resistant melons

Developing virus-resistant melons presents numerous challenges due to the complex interactions between viruses, host plants, and environmental factors. One of the primary difficulties in breeding virus-resistant melons is the evolutionary potential of viruses. Owing to their large population size and short generation time, viruses can rapidly evolve and adapt under selection pressures, including those imposed by resistant host plants (Gallois et al., 2018). This evolutionary adaptability can lead to the emergence of resistance-breaking virus mutants, undermining the effectiveness of resistance genes introduced through breeding programs (Gallois et al., 2018). Additionally, the introgression of traits from Crop Wild Relatives (CWR) during the breeding process can significantly alter the genetic background of melons, potentially affecting the durability and efficiency of resistance traits (Gallois et al., 2018). Traditional breeding methods for virus resistance may have limited effectiveness due to the genetic complexity and variability of virus populations. For instance, resistance in the ‘Freeman cucumber’ (C. melo Group Conomon) is controlled by three recessive genes that reduce virus titer, but this resistance can be overcome by certain strains of the Cucumber Mosaic Virus (CMV) (Shahwar et al., 2024).Moreover, there are currently no commercially available CMV-resistant melon cultivars adapted to western production areas, highlighting the challenge of developing broadly effective resistance (Shahwar et al., 2024). However, there was heterogeneity in the responses of some accessions to some strains. For example, resistance in the Korean accession ‘Sonwang charmi, PI 161375’ showed partial resistance to B20.2 and total resistance to isolate M373 (Essafi et al., 2008). And it was identified a QTL linked to CMV resistance on linkage group XII in the exotic melon variant “Sonwang Charmi” (Essafi et al., 2008; Shahwar et al., 2024).Breeding for resistance also involves balancing multiple desirable traits. Melons need to not only resist various viruses but also maintain high Brix content, desirable fruit flesh color, appropriate fruit shape, and other agronomically acceptable traits (Bachiava et al., 2018). The introduction of multiple virus resistance while minimizing deleterious traits is a complex task, as evidenced by the effort to develop melons resistant to multiple viruses such as WMV, Zucchini Yellow Mosaic Virus (ZYMV), and CMV (Bachiava et al., 2018).

Despite these challenges, advances in breeding techniques and the use of molecular markers linked to QTL offer promising avenues for developing virus-resistant melon lines (Bachiava et al., 2018). However, the economic returns of resistance breeding must be weighed against the costs, especially given the limited success of traditional breeding under high virus pressure (Kenney et al., 2020). Employing a combination of breeding methods, such as recurrent selection to develop base populations with general adaptation and desirable fruit characteristics, may help achieve the complex objectives of melon breeding programs (Samantara et al., 2022).

## Contributions to sustainable agriculture and environmental conservation

Sustainable agricultural operations not only yield positive impacts on the environment, animals, and people but are also imperative for long-term food security and environmental health. One of the central themes of sustainable agriculture is the conservation of resources critical for agricultural productivity, which includes maintaining the integrity of soil and managing pest populations effectively (Thudi et al., 2021). In the context of melon cultivation, virus management is primarily achieved through the control of insect vectors using insecticides. However, this method has proven to be only marginally effective in preventing virus transmission, and in some cases, it can exacerbate the spread of viruses by encouraging vector visitation to infected plants (Kenney et al., 2020).

Consequently, breeding melons that are resistant to viruses like the Melon leaf curl New Delhi virus can significantly reduce the reliance on insecticides, thereby minimizing environmental pollution and promoting ecological balance (Kesh and Kaushik, 2021). Furthermore, managing insect pests through sustainable practices, such as the timely destruction of volunteer melons between cropping cycles, helps to reduce the source of virus inoculum. Techniques like disking or applying burn-down herbicides to emerging volunteer plants are practical approaches to control pest populations without excessive chemical use (Castle et al., 2017). These strategies not only enhance yield safety and fruit quality but also support the broader goals of sustainable agriculture by preserving biodiversity and promoting the health of agricultural ecosystems (Kesh and Kaushik, 2021; Thudi et al., 2021).

## Future directions in melon breeding for virus resistance

**Figure 1:**
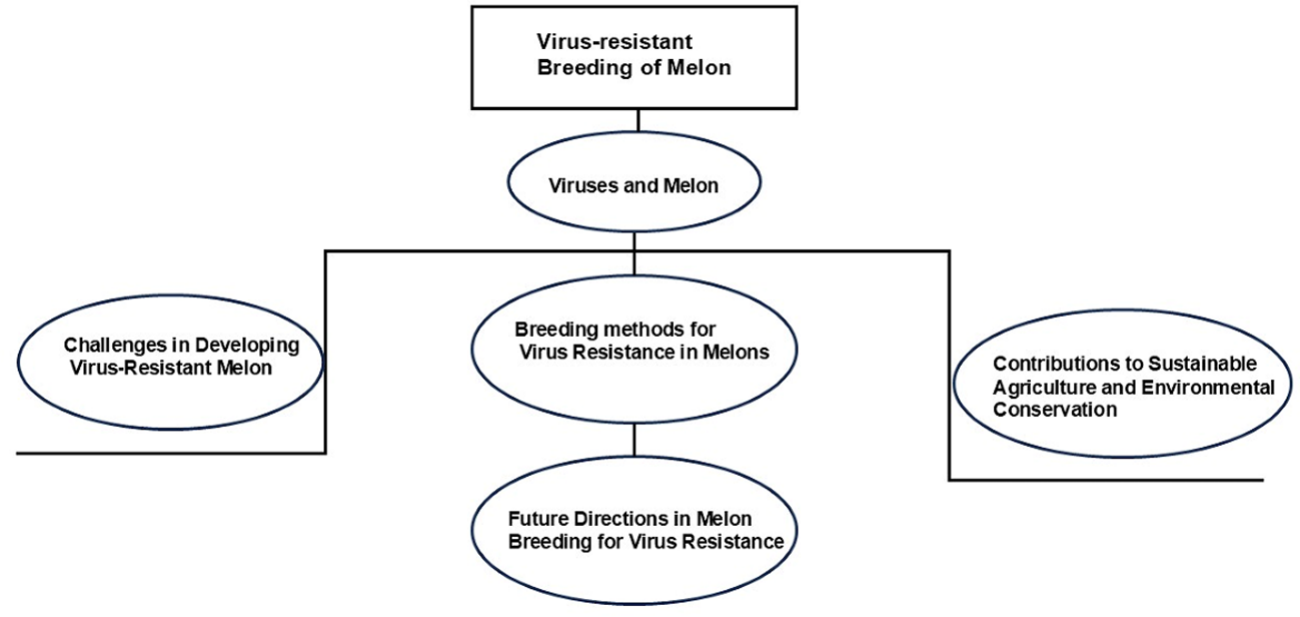
Outline of the summary content.

Future efforts in melon breeding for virus resistance are set to benefit from a combination of advanced genetic tools and novel breeding strategies (**Figure 1**). With the recent sequencing of the melon genome, researchers now have access to a wealth of genetic information that can facilitate the identification and incorporation of virus resistance genes into new melon varieties (Argyris et al., 2015; Shahwar et al., 2023). The melon genome comprises approximately 375 Mbp and 27,427 protein-coding genes, offering a substantial resource for genetic studies and breeding programs (Shahwar et al., 2023).

1) Strengthen the collection and evaluation of virus - resistant germplasm, as it is the fundamental basis for breeding. This includes searching for diverse melon varieties with potential virus -resistance traits from different regions and ecological environments.

2) Utilize advanced genetic engineering techniques to introduce specific virus - resistant genes into melon genomes, precisely targeting the most harmful viruses.

3) Develop molecular markers associated with virus resistance. These markers can be used in marker - assisted selection during the breeding process to quickly identify and select melon plants with desired resistance traits. These markers can significantly enhance the efficiency of selecting resistant traits during the breeding process (Collard and Mackill, 2007; Bachiava et al., 2018). Moreover, integrating DNA markers with traditional breeding methods allows for more precise characterizations of genetic resources, thereby aiding breeders in selecting optimal parent lines (Collard and Mackill, 2007). The 100 Melon Genome project exemplifies the collaborative efforts being undertaken to advance melon breeding. This project, coordinated by Dr. Rob Dirks from Rijk Zwaan Breeding BV and involving multiple breeding companies and academic institutions, aims to harness genetic expertise and extensive germplasm collections to develop improved melon varieties with enhanced resistance to viruses (Palumbo, 2020).

4) Focus on breeding melons with multiple - virus resistance. Instead of just targeting a single virus, aim to create varieties that can withstand several common and economically significant viruses simultaneously. The application of genome-wide association studies (GWAS) has been instrumental in identifying genes or QTL associated with key traits related to domestication, such as fruit mass and quality, as well as disease resistance (Siskos et al., 2022). These insights are invaluable for breeding programs focused on developing multi-virus-resistant melon lines that do not compromise on other agronomic traits. However, the path forward is not without challenges. Melon varieties bred for extended shelf life, for example, are often more sensitive to environmental variations and may require specific management practices to ensure their resilience to virus pressure (Kenney et al., 2020). Moreover, integrating resistance traits into commercial varieties while minimizing associated deleterious effects remains a key objective for breeders (Bachiava et al., 2018).

5) Collaborate with international research institutions to share data and resources related to melon virus resistance breeding. This can accelerate the development of new resistant varieties by leveraging global expertise.
